# Successful aging is environmentally friendly aging: a lifespan perspective of successful aging in the context of climate change

**DOI:** 10.1093/geront/gnag153

**Published:** 2026-07-11

**Authors:** Selma Korlat, Liat Ayalon, Anna E Kornadt, Karl Pillemer, Klaus Rothermund, Jana Nikitin

**Affiliations:** Department of Developmental and Educational Psychology, Faculty of Psychology, University of Vienna, Vienna, Austria; Louis and Gabi Weisfeld School of Social Work, Bar Ilan University, Ramat Gan, Israel; Department of Behavioral and Cognitive Sciences, Faculty of Humanities, Education and Social Sciences, University of Luxembourg, Esch-sur-Alzette, Luxembourg; College of Human Ecology, Cornell University, Ithaca, New York, United States; Department of Psychology, Friedrich Schiller University of Jena, Jena, Germany; Department of Developmental and Educational Psychology, Faculty of Psychology, University of Vienna, Vienna, Austria

**Keywords:** Successful aging, Climate change, Lifespan, Climate action, Resilience

## Abstract

Aging in the 21st century is marked by the distinct challenge of climate change that affects not only today’s older adults but also future generations who, if fortunate, will themselves grow old. In this article, we propose that enhancing successful aging among the current cohort of older adults and preparing younger generations to age successfully is inherently aligned with the pursuit of a more sustainable future. This alignment can be achieved through the adoption of individual pro-environmental health-promoting behaviors, active engagement in climate-related action, and the fostering of psychological adaptation and resilience across the life course. We argue that in the context of the climate crisis, successful aging presents not only a personal or public health goal, but also a potential pathway toward sustainability, intergenerational solidarity, and collective resilience. This article provides an overview of negative implications of climate change for the respective dimensions of successful aging and discusses possible developmental, social and environmental interventions for each dimension throughout the lifespan.

As early as 44 BCE, old age was described by Cicero in his work *De Senectute* not as a burden or decline, but as a natural and even advantageous phase of life, if lived wisely and virtuously. This reflects the idea that, if we are to age well—both as individuals and as a society—we must actively invest in the aging process while also acting in moral and socially responsible ways. This idea has become even more important in the face of climate change, which threatens safety and contributes to worsening environmental and societal conditions. Although every period in human history has faced its own challenges, climate change creates a notably different aging trajectory compared to past generations. At the same time, the global growth in the older population makes the relationship between population aging and climate change increasingly relevant to a growing number of people. Thus, aging successfully in the context of climate change concerns not only today’s older adults, but also younger people—including children and future generations yet to be born—most of whom will one day grow old.

However, as noted by the World Health Organization (2022, p. 3): “…aging and healthy longevity for most people–now and in the future–will not be possible without a healthy planet.” This challenge is further compounded by the fact that the consequences of current human activities—particularly greenhouse gas emissions from fossil fuel combustion, deforestation, and industrial processes—will become increasingly prevalent and more damaging in the decades ahead (Intergovernmental Panel on Climate Change, 2023). Therefore, planning for successful aging in the context of climate change, while important at present, will be even more critical for future cohorts of older people. The choices made today at both individual and system levels will shape aging, climate, and societies for generations, making urgent action essential.

This article contends that it is possible to promote successful aging while simultaneously contributing to mitigation of climate change, engaging in climate-related action as a form of response, and fostering psychological adaptation and resilience. We propose that such an approach can contribute to promoting well-being and healthy longevity of current and future generations of older adults, as well as planetary health and environmental protection.

We would note at the outset that this article should not be misunderstood as advocating a naïve perspective that successful aging, or combating climate change, is simply a question of healthy behavior, social engagement, and resilience, thus putting the burden explicitly or implicitly on the aging individual. We recognize that climate change creates situations in which successful aging may become impossible for some older people, regardless of their efforts and resources. In fact, this is already the case for many older people in the Global South, as well as individuals in problem areas that are disproportionately affected at present by the consequences of climate change. The goal of this article is instead to emphasize the mutual interrelationships between climate change and successful aging, proposing that fostering conditions for successful aging is critical to combat climate change effects, requiring a joint effort of all generations.

## Successful aging in the context of climate change

As the number of older adults increases, there is a pressing need to focus on how they can remain as functionally capable and healthy as possible. The urgency of this need has dramatically increased in the context of increasingly complex climate-related challenges. Successful aging is captured in Rowe and Kahn (1997)’s model as a process characterized by (a) a low risk of disease and disability, (b) high physical and cognitive functioning, and (c) active engagement with life. The concept of successful aging is closely related to the term *healthy aging*, defined as encompassing the dynamic process of developing and sustaining functional ability and intrinsic capacity to promote well-being in older age (World Health Organization, 2021). Similarly, the successful aging concept overlaps with the concept of *sustainable aging—*the maintenance of the functional capacity necessary for well-being in old age in contexts experiencing crises, such as climate change and war (Asiamah, 2023). Despite well-documented critiques (Martinson & Berridge, 2015), all these concepts revolve around the same fundamental idea: promoting quality of life and an extended “healthspan,” rather than merely increasing years of life (“lifespan”; Di Ciaula & Portincasa, 2020).

Rather than contrasting competing aging paradigms, our goal in this article is to use an established aging framework to illustrate how environmentally friendly behavior may simultaneously support aging and climate change mitigation. We therefore rely primarily on the successful aging model (Rowe & Kahn, 1997) because its three core pillars provide a clear and intuitive structure for conceptualizing how environmentally friendly behaviors may support aging outcomes across multiple domains. Importantly, our perspective remains closely aligned with healthy and sustainable aging approaches in emphasizing functioning, well-being, and person–environment interactions across the lifespan (Menassa et al., 2023).

We propose that successful aging is inheritably linked to environmentally friendly behavior. In relation to the first two pillars—a low risk of disease and disability and high physical and cognitive functioning—we suggest that individual pro-environmental behaviors and sustainable lifestyle choices may promote personal health and well-being across the lifespan. In doing so, they reduce the risk of disease and disability and maintain physical and cognitive functioning, while simultaneously reducing environmental harm and contributing to climate change mitigation. Regarding active engagement in life, we propose that socially engaged aging includes active participation in collective climate action. Such action both fulfills the criterion of engagement with life and supports the societal transformations needed to address climate change. In addition to physiological and social components of successful aging, more recent research has highlighted the importance of its psychological factors, identifying psychosocial components as even more integral components of successful aging than physiological components (Cosco et al., 2013). Similarly, a more recent meta-analysis of correlates of successful aging found psychological adaptation in later life to be the most influential domain of successful aging in old age (Kim & Park, 2017). Therefore, we propose psychological resilience—having a positive outlook on both future and aging in the context of climate change, feeling agentic and empowered—as a crucial prerequisite for the first two pillars and an important psychological resource for adaptation that may also catalyze individual and collective change in the context of climate change.

Although our focus is on later life, we consider these dimensions as relevant across the entire lifespan. We argue that enhancing successful aging in currently older adults and preparing younger generations to age successfully is inherently aligned with the pursuit of a more sustainable future. In this article, we begin by examining the links between climate change and health of older and aging adults. Next, we discuss the relationship between climate change and social engagement. We then explore the role of psychological resilience in the face of climate change. Within each section, we first highlight negative implications of climate change for the respective dimension of successful aging and then discuss possible developmental, social and environmental interventions to counteract and mitigate these effects across the lifespan.

### Adopting individual pro-environmental behaviors: fostering health span and contributing to climate change mitigation

#### Effects of climate change on health and physiological component of successful aging

In 2024, the Lancet Countdown on Health and Climate Change reported record-breaking threats to global health and well-being, with two-thirds of key climate–health indicators reaching their highest levels (Romanello et al., 2024). As one of the main findings, the Lancet Countdown report identified that heat-related mortality among people older than the age of 65 rose by an unprecedented 167% compared to levels in the 1990s (Romanello et al., 2024), positioning older adults as a distinct at-risk group within the broader landscape of climate change impacts (Khalil et al., 2025). A recent systematic review of health indicators in the context of climate change and aging confirmed that most climate-related phenomena (such as extreme and abnormal temperatures, strong winds, sea temperature variability, droughts, air pollution from wildfires) are linked to increased risks of all-cause and cause-specific mortality, as well as various morbidity outcomes in older adults, with cardiovascular and respiratory diseases identified as the most common causes of hospitalization (Figueiredo et al., 2024).

The future outlook raises even greater concern. It has been estimated that in the absence of effective mitigation measures, a 264% (range 117.5% to 427.3%) increase of heat-related mortality will occur by 2080, as compared to 1980 (Li et al., 2016). Increasing evidence suggests that exposure to environmental pollution, including early-life exposure, is associated with a higher risk of chronic neurological diseases such as Alzheimer’s disease and related dementias, metabolic conditions including type 2 diabetes and other noncommunicable diseases, as well as sarcopenia and frailty in older adulthood (for a review, see Di Ciaula & Portincasa, 2020). As environmental pollution and climate-related environmental stressors are projected to increase in the future (Intergovernmental Panel on Climate Change, 2023), pollution-related risks for noncommunicable diseases may pose an increasing challenge for healthy and successful aging in future generations. Research indicates that climate change has negative implications for both biological and cognitive aging (for a review of biophysical vulnerabilities of older adults in the context of climate change, see Tipaldo et al., 2024). From a life course perspective, these findings suggest that younger generations will experience the impacts of climate change over a longer portion of their lives, making climate a direct and ongoing influence on their ability to age successfully. Taken together, the evidence for the growing negative effects of climate change in later life underscores the importance of initiating interventions early in life to foster long-term health, physical and functional capacity.

#### Possible mitigating measures throughout the life span

While interventions that promote health and healthy aging have different foci depending on the life phase—with growth and development being the focus in childhood, optimization of health in adulthood, and prevention of decline in older age—the main strategies across the whole lifespan include balanced and healthy nutrition, active life-style, social engagement (Cocchi et al., 2025), as well as sufficient sleep, cognitive stimulation, and avoidance of harmful behaviors (Seals et al., 2016). Some of these lifestyle habits align with, or complement, pro-environmental behaviors, thereby mitigating climate change while simultaneously strengthening resilience to climate-related health threats. For example, biking and walking have positive individual health impacts related to physical activity, while also reducing carbon emissions and air pollution (Dabelko-Schoeny et al., 2024). Similarly, adopting a plant-based or low-meat diet supports cardiovascular and metabolic health while significantly reducing the environmental footprint and resource demands (Viroli et al., 2023). Moreover, a recent review (Albarracin et al., 2024) identified similar mechanisms underlying successful behavior change in both health-related and pro-environmental domains. Interventions targeting emotions, behavioral skills, attitudes, injunctive and descriptive norms, as well as the use of monitors, reminders, and material incentives, showed small effects, whereas habit-promoting interventions, habit-reversal training, and social support showed medium-sized effects. Structural access-related interventions (e.g., to vaccines or recycle bins) have been found to have large effects on adopting healthy and individual pro-environmental behaviors (Albarracin et al., 2024). These findings suggest that health-promoting and pro-environmental behaviors may rely on partly shared psychological and contextual determinants, implying that strategies effective in one domain may also be applicable in the other.

Social interventions such as providing children with greater opportunities, skills, and recognition for involvement in their classrooms and families have been shown to be associated with better health maintenance behavior, mental health, and overall adult health and success more than 20 years later (Kosterman et al., 2019). Similarly, peer interactions and family involvement emerged as critical components in enhancing motivation and fostering behavioral change regarding dietary and physical activity among adolescents (Maycroft et al., 2026), as well as in promoting pro-environmental behaviors (Wallis & Loy, 2021). At the same time, a systematic review on the role of social factors in successful aging, consisting predominantly of longitudinal studies, demonstrated that social factors earlier in life substantially shape successful aging outcomes (Takács & Nyakas, 2022). Taken together, to effectively promote health and pro-environmental behaviors, and support successful aging all lifestyle factors mentioned in this section should be implemented for children and their families from the earliest years of childhood (Dalal et al., 2022; Evans et al., 2018).

In the context of environmental interventions, the development of age-friendly but also *longevity-ready* cities (Wang et al., 2021) emerges as a key strategy. Urban planning approaches that prioritize the expansion of green spaces can facilitate both climate mitigation and adaptation, while offering substantial health co-benefits. These include reduced exposure to air pollution, local cooling effects during heat events, stress reduction, and increased opportunities for physical activity and recreation (White et al., 2023). Moreover, spending time in natural environments—through activities such as gardening, hiking, or forest bathing—has been associated with lower stress levels, better mental health, and improved immune functioning, while also fostering environmental connectedness and a sense of environmental stewardship among older adults (Korlat & Nikitin, in press).

For older adults already navigating age-related changes, these interventions can help maintain functional independence and cognitive vitality in the face of increasing environmental stressors. More critically, when adopted early and sustained throughout the life course, these behaviors create a foundation for successful aging that is inherently adaptive to climate challenges while, at the same time, contributing to climate mitigation.

### Social engagement and response to climate change

#### Effects of climate change on the social component of successful aging

Climate change effects, such as extreme weather events and environmental disruptions, can limit opportunities for social interactions and integration among older people, often intensifying existing vulnerabilities. Displacement and loss resulting from climate-related disasters disrupts established social networks, leading to social fragmentation and a sustained sense of disconnection (Ayalon, 2025). Moreover, social isolation is a risk factor for negative effects; for example, heat waves are linked to increased mortality particularly among socially isolated older people (Klinenberg, 2015; Orlando et al., 2021).

From an intergenerational perspective, older adults are sometimes perceived as responsible for climate change or obstructing necessary societal transformation, which may in turn foster social exclusion and intergenerational tensions (Ayalon & Roy, 2023). Older individuals may also be viewed as out of touch or incapable of fully grasping science behind the urgency of the crisis. Younger generations, in contrast, experience the burden of responsibility, feeling that they are expected to rectify the damage caused by previous generations (Stollberg et al., 2024). Despite their efforts to raise awareness and advocate for action, young people may feel marginalized in policymaking, discredited, or dismissed, perceived as alarmist or naïve, even while fighting for their legitimate stake in the future (Ayalon et al., 2023). Such experiences can undermine their well-being, positive identity, social integration, and opportunities for engagement (Jones, 2023), thereby limiting social resources that can support successful aging (Takács & Nyakas, 2022).

#### Possible mitigating measures throughout the life span

Generativity represents a powerful motivator in the climate context, especially for older adults, as they are developmentally driven by concern for future generations and a desire to share their experience and knowledge (Diehl, 2022). An experimental study found that messaging focused on legacy concern significantly increased pro-environmental behavioral intentions and donations to environmental organizations across younger and older age groups, compared to a control condition (Wickersham et al., 2020). Similarly, a recent mega-study conducted across 63 countries found that policy support was strengthened by writing a letter to a future-generation member (Vlasceanu et al., 2024).

For generativity to be meaningfully expressed, it is essential to create avenues for intergenerational dialogue and collaboration. Climate change has been characterized as an intergenerational challenge that requires intergenerationally-focused behavioral solutions (Law et al., 2025). This focus requires that the contributions of both younger and older people be welcomed, recognized, and valued, and that actionable pathways be established to implement the changes they propose (Kennedy & Gislason, 2022). Research highlights the potential of intergenerational solidarity (Ayalon et al., 2023), emphasizing the importance of centering messages of inclusivity and cooperation—particularly as an alternative to polarizing or accusatory narratives—in order to build a shared future that benefits all generations (Roy & Ayalon, 2024). An experimental study confirmed that interest in the younger generation’s opinion and giving them voice, offering reparation, and acknowledgment of the younger generation’s achievements and capabilities in fighting climate change coming from older activists, compared to younger activists and a neutral condition (non-empowering text describing typical leisure time activities of the younger), increases intergenerational reconciliation (Stollberg et al., 2024).

On the other hand, inclusion of older adults in climate change mitigation efforts can inspire and motivate younger generations and serve as a powerful call to action (Katey & Zanu, 2024). Moreover, opportunities to express generativity and social inclusion are also important goals for many older adults, leading to positive effects on well-being (e.g., Lodi-Smith et al., 2021; Moieni et al., 2020). Therefore, for many older individuals, activism and social engagement in the context of climate change can be a vehicle to act in accordance with developmental tasks, personal values, social role and a sense of moral responsibility toward future generations, enhancing psychological resilience and life satisfaction (Pillemer et al., 2022), promoting their successful aging.

Other effective strategies to foster engagement among older adults, include tailored communication approaches—such as the use of appropriate language, visual materials, and emotionally resonant or culturally relevant messaging (Ni et al., 2025)—combined with efforts to remove barriers to volunteering (Pillemer et al., 2022). However, Vlasceanu et al. (2024) found that the effectiveness of behavioral climate interventions significantly varies depending on the target audience and specific behaviors. These findings underscore the importance of further investigation into how interventions can be tailored across different age groups, taking into account possible barriers that prevent older adults from becoming engaged, while also accounting for intersections with other axes of social differentiation (gender, socioeconomic status, geographical position, etc.).

In terms of environmental factors, the natural environment offers a valuable setting for intergenerational social cohesion and community interaction (Finlay et al., 2015), as well as pro-environmental behaviors across the lifespan (Korlat et al., 2025), while also providing physical benefits such as cooling and shade. These features become increasingly important as climate change effects, such as heat, limit opportunities for social engagement and, in turn, pose risks to successful aging. Similarly, digital tools and social media platforms have shown potential in mobilizing individuals and fostering resilient, climate-conscious communities (Lestari et al., 2025), serving as valuable means of intergenerational interaction and engagement when in-person contact is limited due to climate-related events such as heatwaves or other extreme weather conditions.

By positioning both younger and older adults as valued contributors to climate solutions rather than passive recipients of climate impacts, this intergenerational framework reframes aging in the climate era as an opportunity for continued meaningful engagement across the lifespan, thereby supporting the psychological and social dimensions of successful aging even as environmental challenges intensify.

### Psychological resilience and adaptation to climate change

#### Effects of climate change on the psychological component of successful aging

In the context of climate change, psychological components of successful aging—such as perception of the aging process, life satisfaction and purpose in life (Kim & Park, 2017) gain increasing significance, as they are particularly threatened in the current and future climate-scenarios. This risk to successful aging can be increased in future generations of old adults, as climate change significantly affects an individual’s sense of purpose, creates uncertainty about the future (Jones, 2023), and elicits strong negative emotional responses—such as anxiety, grief, and overwhelm, particularly among younger populations (Poortinga et al., 2023). Therefore, it is important to evaluate how individuals throughout the life course envision their aging and their future as older adults in a world increasingly shaped by climate change.

Similarly, research with adolescents shows that climate change anxiety can weaken future self-continuity—how connected they feel to their future selves—making it harder for young people to envision and plan for their future, which in turn reduces their motivation for pro-environmental behaviors (Qin et al., 2024). This suggests that when individuals feel less connected to their future selves due to the unpredictability and threats posed by climate change, they may struggle to maintain a stable sense of identity and purpose, and may become less likely to engage in long-term, future-oriented actions. On the other hand, although older generations tend to report less intense negative emotions related to climate change compared to younger individuals (Poortinga et al., 2023), old and very old adults (Ayalon, 2024) do experience emotional distress in response to the changing climate which can negatively affect their ability to age successfully.

#### Possible mitigating measures throughout the life span

We propose promoting a more hopeful and empowered views of aging in the context of climate change among both older and younger generations as a pathway toward achieving both successful aging and a more sustainable future. People with more positive views of their future selves as older adults are more likely to prepare for age-related changes, engage in healthier behaviors, and experience more positive life events, while negative views can lead to less preparation and more negative outcomes (Kornadt et al., 2015; Voss et al., 2017; Wurm et al., 2017). Imagining a dire future as an older person impacted by climate change and associated negative developments can thus lead to actual negative individual outcomes. A recent meta-analysis suggested that positive age-related beliefs, defined by strength and psychological growth, foster a more optimistic outlook and adaptive coping strategies, while negative views of aging can promote feelings of worthlessness and discourage active engagement in life (Trică et al., 2024). Similarly, a recent study that systematically tested 17 interventions that targeted psychological mechanisms to motivate climate action found that interventions that targeted future thinking (e.g., imagining experiencing a preventable negative future that could occur due to climate change or imagining experiencing a positive future) were the most effective for broadly motivating individual actions (e.g., driving less, eating vegetarian meals) and collective actions (e.g., donating, volunteering; Sinclair et al., 2025). Thus, a positive, hopeful and empowered outlook on aging and the future may serve as a crucial source of motivation to act while simultaneously aging successfully, even in the face of adversity (Sun & Li, 2025).

This idea reflects the concept of resilience, which encompasses adaptive mechanisms that play a crucial role in maintaining and enhancing quality of life in old age (Bowling & Iliffe, 2011). Resilience is most generally defined as the ability to absorb shocks, self-organize, and adapt to changing circumstances (Haq & Gutman, 2014, 2020). However, within social-ecological systems, resilience is understood as the capacity of living beings to anticipate change and actively shape future pathways in response to disruptions or disasters (Vázquez-González et al., 2021), a process closely aligned with the concept of proactive coping (Aspinwall & Taylor, 1997). In this sense, resilience goes beyond mere adaptation and acceptance—it involves proactive engagement with foreseeable challenges, such as those posed by climate change. Rather than reacting passively, resilient individuals and communities are those who prepare for, respond to, and influence the trajectory of change in constructive ways (Tanner et al., 2015). This perspective should be integrated into the self-concept from early on in life, so that people grow up aware of their lasting power to shape their world as active agents—not only in youth, but also throughout the aging process and well into later life. This approach aligns with the concept of a “*resilience reserve*,” which defines resilience as the sum of processes and resources that protect against and compensate for challenges or the effects of adversity (Brinkhof et al., 2025; Cosco et al., 2017).

However, when adverse situations (e.g., extreme weather events) are unavoidable, it is equally important to cultivate the capacity to cope effectively, absorb shocks, and maintain a sense of stability and purpose in the face of difficulty. Especially with regard to climate change, it is important to identify realistic goals and actions, and to adapt one’s aims to what is feasible, rather than getting stuck with unrealistic ideals that can become a source of chronic frustration that will ultimately erode one’s motivation to engage because every action will seem futile. A recent study examining age differences in emotional well-being among adults exposed to hurricane-related stressors found that older individuals—particularly those who had directly experienced such adversities—were the least likely to report symptoms of depression and showed fewer signs of post-traumatic stress disorder (Strough et al., 2024). These findings highlight that older adults might be able to successfully cope with such disasters, and psychological resources and coping strategies may represent key components for maintaining successful aging in an era of climate uncertainty, by enabling aging individuals to remain engaged, adaptive, and future-oriented despite ongoing environmental change.

## Discussion

Scientific work on successful aging centers on identifying the factors and conditions that increase the potential of older adults and uncover ways to reshape nature and the experience of aging (Estebsari et al., 2020). The nature of aging will inevitably evolve in response to global demographic shifts and the increasing threat of climate change. It is therefore increasingly important to consider how humans—both individually and collectively—can follow Cicero’s advice to age “wisely and virtuously” in the 21st century. In this article, we propose that leveraging the core components of successful aging—physiological, social, and psychological—can contribute to a more sustainable future, as the efforts to promote successful aging align closely with the Sustainable Development Goals (Prina et al., 2024), offering a mutually reinforcing path toward individual and planetary health and well-being.

We have highlighted the importance of building resources for old age throughout the life course by a combination of measures targeting the three core facets of successful aging: (a) Promoting and maintaining health through individual pro-environmental behaviors, (b) accumulating social capital through lifelong engagement in civic and climate action while fostering intergenerational solidarity, and (c) building psychological and resilience capacity through cultivating positive views of aging throughout the lifespan and empowering (future) older people. Moreover, the three facets are closely interconnected and mutually reinforcing. Psychological resilience and adaptive coping, for instance, have been shown to foster engagement in climate action (Bingley et al., 2022; Sinclair et al., 2025). At the same time, a systematic review of interventions aimed at reducing climate anxiety identified climate engagement, as well as social support and community building as effective strategies for alleviating climate-related distress (Bingley et al., 2022), thereby further strengthening psychological resilience. In parallel, psychological and social resources facilitate the adoption and maintenance of health-promoting and preventive behaviors (e.g., Yildirim & Arslan, 2022) while better physical and mental health may, in turn, increase individuals’ capacity and resources to remain socially engaged and psychologically resilient. Moreover, a large-scale “intervention tournament” study has shown that interventions that targeted two or more mechanisms are most effective in motivating action (Sinclair et al., 2025).

However, it is important to acknowledge the complex interrelations between different factors contributing to successful aging and climate change, as well as substantial interindividual differences and inequalities among older adults in terms of resources, opportunities, health, and living contexts. For some older adults, maintaining contact with family, friends, and local communities through fully sustainable means of transportation may not be feasible. In such cases, the use of fuel-based transportation (e.g., cars or airplanes) may be necessary to preserve important social ties and potentially climate action, particularly in rural areas. Similarly, heating and cooling technologies are especially important for older adults, given their heightened vulnerability to morbidity and mortality during heat waves and cold spells (Figueiredo et al., 2024). Although the use of such technologies may increase greenhouse gas emissions, they remain essential for preserving health and functional independence. Similarly, older adults often rely more heavily on medical treatments and healthcare services, which contribute substantially to global greenhouse gas emissions (Haas et al., 2023). However, these services are indispensable for maintaining health and quality of life.

Similarly, post-retirement migration may both facilitate and constrain successful aging. On the one hand, retirement migration may improve well-being by enabling older adults to move closer to family members, access more favorable climates, or live in areas with lower costs and higher perceived quality of life. On the other hand, relocation may disrupt established social networks, reduce continuity of healthcare, or increase vulnerability if older adults move to regions with limited support systems or warmer climate (Haan et al., 2025). These examples further illustrate that pathways toward successful aging are context-dependent and shaped by broader social, environmental, and structural conditions.

These examples highlight that the relationship between successful aging and environmentally sustainable behavior is not always straightforward and may involve trade-offs between individual well-being and environmental impact. Therefore, rather than placing responsibility solely on individuals, it is essential to develop system-level infrastructures and policies that enable older adults to age healthy, resilient and remain socially connected while minimizing environmental burden wherever possible.

## Implications

The synergy between successful aging and environmental stewardship offers far-reaching social, economic, and systemic benefits (Rowe, 2024). In addition to its direct impact on human health and mortality, climate change imposes a substantial economic burden, with budgetary pressures on the private and public (e.g., healthcare) sectors (Haas et al., 2023). Thus, implementing proactive interventions on successful aging can reduce long-term expenditure on treatment and care. Moreover, the healthcare sector itself is a significant contributor to greenhouse gas emissions in developed countries—a factor frequently overlooked (Haas et al., 2023), creating a vicious cycle of pollution and impact on health and aging. Thus, successful aging might constitute a solution to the main burdens imposed by population aging both on individuals and systems (Lee et al., 2020). However, it is important to note that strategies to achieve successful aging in the context of climate change require coordinated efforts at the individual, community, and society level.

It is also important to note that effects of climate change are different in different regions and different populations. Vulnerable populations that are simultaneously less responsible for climate change, and less resilient to climate stressors because of health and economic disparities are more affected—such as people in low-income and disadvantaged countries (Limaye, 2022). The most vulnerable older people are those with disabilities and/or chronic medical conditions and those living alone or in nursing homes or care facilities, and those lacking resources (World Health Organization, 2022), particularly low-income older adults of color with functional limitations (Carlson et al., 2024). Other intersectional factors like ethnicity, gender and sexual orientation exacerbate disparities (Goldsmith & Bell, 2022). A comprehensive, multi-faceted strategy is needed to address inequalities in the effects of climate change, particularly as they relate to aging. We call for greater attention to the design and implementation of successful aging interventions that are sensitive to regional disadvantages and contextual vulnerabilities, acknowledging the diversity of individual needs and capacities in accessing and utilizing both personal and systemic resources. Addressing this intersection is not only an environmental concern but also a matter of social justice—ensuring that aging populations are protected, empowered, and included in building a resilient, climate-adaptive future (Katey & Zanu, 2024).

## Conclusion

In this article, we have outlined the approaches to promote successful aging in the context of climate change while simultaneously fostering motivation for both individual and collective climate action. When health-promoting and environmentally friendly behaviors are cultivated as lifelong habits, they become embedded in everyday routines and personal identity. In doing so, they contribute naturally to successful aging and an extended *healthspan—*both for the individuals and for the planet.

## Data Availability

This article does not report data and therefore the pre-registration and data availability requirements are not applicable.
